# Answer to a Paper on Phrenology, in the Number for June, Signed Junius

**Published:** 1822-08

**Authors:** Edw. Th. Bond

**Affiliations:** Member of the Royal College of Surgeons.


Art. VII.—*Answer to a Paper on Phrenology, in the Number for June, signed* Junius. By Edw. Th. Bond, Member of the Royal College of Surgeons.

The communication of your correspondent Junius reminds me of the individual, respecting whom it is reported by Addison that he contrived to fill his own snuff-box by soliciting occasionally an auxiliary pinch from whomsoever chance threw in his way. I was not previously aware that epistolary poverty might be remedied in a similar manner; but I have now learned that the only difference between the two modes of proceeding consists in this,—that one is a request made at decent intervals, and the other a kind of peremptory transfer, with scarcely any interval at all. Indeed, it may be said of the author, that he rests from his labour when he ceases from his quotations; like the old man who cannot move without his staff, even though it be a borrowed one; for, satisfied apparently that they fill his sheet, he seems to consider it quite enough that he has removed them to his own pages, as if the bare insertion of them in the midst of error were, in his opinion, a kind of *via regia* towards proving them erroneous. Yet I do not forget that he compliments me, and, if it were in my power, I would be as obliging in return. But the truth is, I am not over anxious to say what I do not think; and can only, therefore, express my regret that he has not afforded me an opportunity of indulging my grateful feelings, except at the expence of my veracity. Plato is one's friend, and Socrates is one's friend; but Truth, as STERNE translates it, is one's sister. Lest, however, I should sink under the consciousness of unrequited obligation, I may console myself with the reflection, that he is not only indebted to me for the chief materials with which he has put together a paper for the London Medical and Physical Journal; but that the observation of mine, that most men are influenced by authority, furnished him with the hint upon which he chose so dignifying an appellation for himself. "Junius" is indeed an imposing sound; and one might have expected that a censor assuming such a title would be as close, clinging, and formidable, as another old man of the sea. But, alas! the possession of the bow of Ulysses did not confer the power to bend it; and the seizing upon the name of the "great unknown" is thus proved to be by no means an appropriation of his talent.

Junius undertakes to convict me of "sophistry and error," but, like Mercutio, he "says more in a minute than he stands to in a month;" for it must be acknowledged that, to fill his paper with extracts from mine, and those, too, not principally concerned in the argument, is rather a novel method of redeeming so formidable a pledge,—but even novelty is no trifling merit. Junius may claim for his ingenuity the "*prima præmia victricis hederæ*;" and I, in submitting to the award, will only observe, that he has, at least, accomplished his undertaking in few words and very summarily; somewhat in his manner of whom the poet says that he

> Flings at your head conviction in a lump,
> And gains remote conclusions at a jump.

Yet the interval of a dozen lines or so, in his communication, seems to have produced a more than corresponding hiatus in the memory, or some other mental quality, of its author; or it would have glared in his face, that to stigmatize that as a "train of sophistry and error," which, with the same penful of ink, he assures us tends to occasion contempt for the doctrine which it opposes, somewhat resembles a contradiction in terms. To say that water will intoxicate, or that butter-milk has the properties of aquafortis, sounds in common ears like an absurdity. But, possibly, the minor task of exposing what he considers the faults of my page may serve as a gentle recreation to prepare Junius for the more arduous attempt to reconcile the inconsistencies of his own.

He transcribes an assertion of mine, that instances at variance with the doctrines of phrenology are daily crossing our path. Why he should give it a place, except from coincidence of opinion, it is hard to tell; unless, indeed, the hint that "none is yet brought forward for public examination," be understood to insinuate a contradiction. Now I repeat what I have said, and leave the conclusion respecting it to the good sense and candour of those who will judge for themselves. For, were I to particularize that Mrs. So-and-so is strictly pious, without any organ of religion; that John *this* and Sarah *that* are rigidly honest, though distinguished by the organ of theft; or that Mr. Such-an-one has great poetical talent, without any corresponding cerebral development; would my conduct be more improper or absurd?—improper, in holding up to notice those to whom such exhibition might be unpleasant; and absurd, in taking pains to tell those to whom I address myself what they are amply able to know without any such puerile and officious information. Suppose I were to say that the English people, generally, make light breakfasts? *Proh Deos!* am I, therefore, obliged to certify how many I may have seen at their tea and bread and butter?

Upon the queries next proposed, it is hardly worth while to pause, and, if it were worth while, would be unnecessary; for Junius himself both puts the question and furnishes the reply. An affirmative, however, may readily be granted, without any fear of endangering the truth; for, although it be allowed that the mind has distinct properties, does it then follow that certain nooks of the skull should be appropriated as the peculiar residence of each? Another such chain of luminous argument would give the very existence of an individual as "damning proof" that he inhabited such and such a chamber in such and such an alley. I should be grieved to kick away a prop from the infirm, yet analogy, upon which Junius seems to bear so much, is frequently no more upon our journey than a "broken reed, upon which if a man lean, it will go into the hand, and pierce it." Let us imagine animals, who had never seen man, to be reasoning by analogy from the figure of the monkey. We hear, they would say, that this unknown creature,

"Who wears sweet smiles, and looks erect on heav'n,"

has no trifling resemblance to our friend here, both in form and habits; and, if their hands are alike, we may infer, from analogy, that the same thing holds good of other parts. Now, the monkey has a long tail, *ergo*, man has a long tail also.

I am accused of being "culpably careful" to conceal that proposition of the system which says that our "actions depend upon the balance of power between our organs;" and Junius may readily stand excused for forgetting that such an article of faith would be rather at variance with the opinion which he quotes from Sir G. M'Kenzie, that, " by observing proportions, we can never pretend to predict actions." Yet it is rather odd that the awkward effect of such conflicting statements should bring to my mind, just now, the clumsy motions of a certain animal, which swims indeed, but always cuts its own throat in the effort. I recollect, it is true, that reason is called the balance of power amongst the whole; and, if there be culpability in not speaking of what I consider unintelligible, I acknowledge myself blame-worthy. Yet, to direct attention to that which I am anxious to conceal, resembles the conduct of Galatea, who hid herself from her lover, yet tittered and pelted him with apples from the corner which she had selected for her retreat. Such balance of power, provided one could stipulate that it should always exist, might possibly, though with no little clumsiness, be adequate to the imagined purpose. An individual, for instance, might be blessed with a prominence of that organ which gives the propensity to kill; but he might, on the other hand, be distinguished by a protuberance of that which confers humanity. In such case, the two would pull different ways, and, neutralizing each other by their respective efforts, would produce delightful consistence of character: yet we should call him but a foolish sportsman who yoked together a swift hound with a sluggish one, or a deer hound with a harrier, in order to produce either uniformity of speed or proper choice of game. But, if reason depend upon the balance of power between the whole, what becomes of it in those awkward cases where the organs which incline to bad actions have greater development than those which incline to good ones? Is the man without reason? or is it independent of the asserted balance, which in such case does not exist? or does it take up its residence with the weaker party, in defiance of the power of the strong? or how is the preponderance remedied? Junius is indignant, also, that I overlook the assistance given " by education, &c. to the side of morals." It is plain, then, even in his opinion, that, under this doctrine, the " side of morals" *requires* assistance! Indeed, it is to be feared that it does; and, if it could even be arranged that it should always meet with it, it is still to be dreaded that morality, like the stone of Sisyphus, would roll back upon our utmost exertions. The " &c." I do not understand. Neither am I capable of imagining how, if one man's conduct be, under any circumstances, such as corresponds with a minor development, that of another can consistently be asserted to be influenced by a major one. But, probably, there are facts in reserve which shall overwhelm the disbelief of the most stubbornly incredulous. It may one day, for instance, be discovered that the magpie, who constructs her nest with an upper half, so as to form a roof, has the architectural organ in twice the proportions possessed by all those meaner birds who content themselves with only a lower one. This once proved, inquirers may be led to investigate whether there be not, in reality, an additional fibre in the cranium for every additional twig between the branches of the hawthorn; and who, that is acquainted with the accuracy of phrenological research, can say where such inquiries shall have an end? It may possibly be ascertained that the foresight of the painstaking ant, who erects storehouses for his winter food, proceeds either from the organ which gives the propensity to build, or from that other which confers the desire to accumulate; while the different habits of the less provident fly, who, in his *gaieté de cœur*, takes no thought for the morrow, may be traced to arise from a total deficiency of both. I once heard of a medical man who said that he "had found the soul:" now, if Junius and he were to lay their heads together, much useful knowledge might be elicited by their united efforts. At all events, when Junius shall have made the microscopical observations to which these hints will, I hope, incite him, the community at large, as well as myself, will, no doubt, be highly obliged by a communication of their important results.

If his assertion be correct, that the last pages of the paper which he has honoured by his notice are "poetical rhapsody signifying nothing," I am, at least, borne out by the kind countenance with which he has favoured me, in affording so promptly a palpable instance of equal deficiency. Be it so, since he has said it. Yet I may just remind him, that want of meaning, with poetical language, is surely better than want of meaning and language both.

In approaching to the "Triste Vale," I may be allowed to regret that I have not the "tongue of the wise to speak a word in season" to him that needs it. If I had, I would whisper to Junius,

"Noli molestus esse omnino literis

Majorem exhibeant ne tibi molestiam."

Then, after an assurance that men of sense willingly "order themselves reverently" before names which deserve honour, I would tell him that an imposing title, like the skin in the fable, is not all that is necessary towards eliciting respect; and, in conclusion, I would gently hint that I do not think him really *a lion*.

*Stoke Newington; July* 10*th*, 1822.

